# Integrative Constraint-Based Modeling and Proteomics Uncover Astrocytic Metabolic Adaptations to the Post-TBI Microenvironment

**DOI:** 10.3390/ijms26136456

**Published:** 2025-07-04

**Authors:** Kelsey A. Wilson, Caiti-Erin Talty, Brian C. Parker, Pamela J. VandeVord

**Affiliations:** 1 Department of Biomedical Engineering & Mechanics, Virginia Tech, 325 Stanger St., Blacksburg, VA 24061, USA; kelseyaw@vt.edu (K.A.W.); caitierin@vt.edu (C.-E.T.); bcparker@vt.edu (B.C.P.); 2 School of Biomedical Engineering and Sciences, Virginia Tech, 325 Stanger St., Blacksburg, VA 24061, USA; 3 Veterans Affairs Medical Center, 1970 Roanoke Blvd, Salem, VA 24153, USA

**Keywords:** traumatic brain injury, flux balance analysis, mitochondrial dysfunction, proteomics, astrocyte metabolism

## Abstract

Traumatic brain injury (TBI) is a major neurological condition affecting millions of individuals each year. Mild TBI (mTBI) manifests differently, with some individuals experiencing persistent, debilitating symptoms while others recover more rapidly. Despite its classification as “mild,” mTBI leads to both short- and long-term neurological effects, many of which occur due to functional changes in the brain. TBI-induced environmental changes within the brain play a critical role in shaping these functional outcomes. The importance of astrocytes in maintaining central nervous system (CNS) homeostasis has been increasingly recognized for their pivotal role in the brain’s response to TBI. Previous studies showed significant TBI-associated metabolic dysregulations. Therefore, we sought to analyze how astrocytes might adapt to persistent metabolic stressors in the post-injury microenvironment and identify injury-induced shifts occurring in vivo that may contribute to chronic metabolic dysfunction. We used an astrocyte-specific genome-scale metabolic model that allowed for the input of biologically relevant uptake rates corresponding to healthy astrocytes to analyze how the activity of metabolic pathways differed in hypoxic and acidic conditions. Additionally, these fluxes were integrated with mass spectrometry-based proteomics from male Sprague-Dawley rats subjected to mTBI to identify chronic adaptive neural responses post-injury. Comparison of modeled metabolic fluxes and experimental proteomic data demonstrated remarkable alignment, with both predicting significant changes in key metabolic processes including glycolysis, oxidative phosphorylation, the TCA cycle, and the Pentose Phosphate Pathway. These overlapping signatures may represent core survival strategies, offering insight into metabolic priorities and potentially serving as biomarkers of injury adaptation or recovery capacity.

## 1. Introduction

Traumatic brain injury (TBI) is the most common form of neurological disorder, with the majority of cases resulting from motor vehicle crashes, unintentional head impacts, and falls. Approximately 27–69 million individuals globally are affected by TBI each year [1,2]. In the United States, TBI-related deaths account for over 2% of all emergency department visits and deaths annually [3]. While over 80% of TBIs can be classified as mild and do not typically result in emergency department visits, they can lead to long-term complications including memory loss, emotional disorders, and the onset of neurodegenerative diseases such as Alzheimer’s and Parkinson’s [4,5,6,7,8]. These clinical outcomes are likely a consequence of environmental changes induced by TBI within the brain, many of which resemble those seen in ischemic injury [9,10,11,12,13,14,15]. A reduction in oxygen within the brain is a commonly observed environmental consequence of TBI [9,10,11,16,17,18,19,20]. Veenith et al. used PET imaging to demonstrate the presence of hypoxic lesions in the brain caused by micro- and macrovascular collapse following TBI [11]. Others have shown reductions in arterial blood oxygen saturation, leading to prolonged inflammation and worse clinical outcomes [16,17]. Clinical data has corroborated reductions in _P_O_2_ in the brain following TBI and associated it with exacerbated reductions in brain tissue pH, indicating acidosis [9,10]. Acidosis is a result of reduced cerebral blood flow due to damaged vasculature as well as functional alterations caused by hypoxia [9]. Low brain tissue pH is correlated with poorer clinical outcomes and has been shown to increase reactive oxygen species generation, induce cellular swelling, and dysregulate metabolism in models of ischemia [9,10,12,13,14,15]. Understanding the fundamental biochemical response has recently become a focus to improve diagnostic and treatment strategies following TBI.

**Figure 1:**
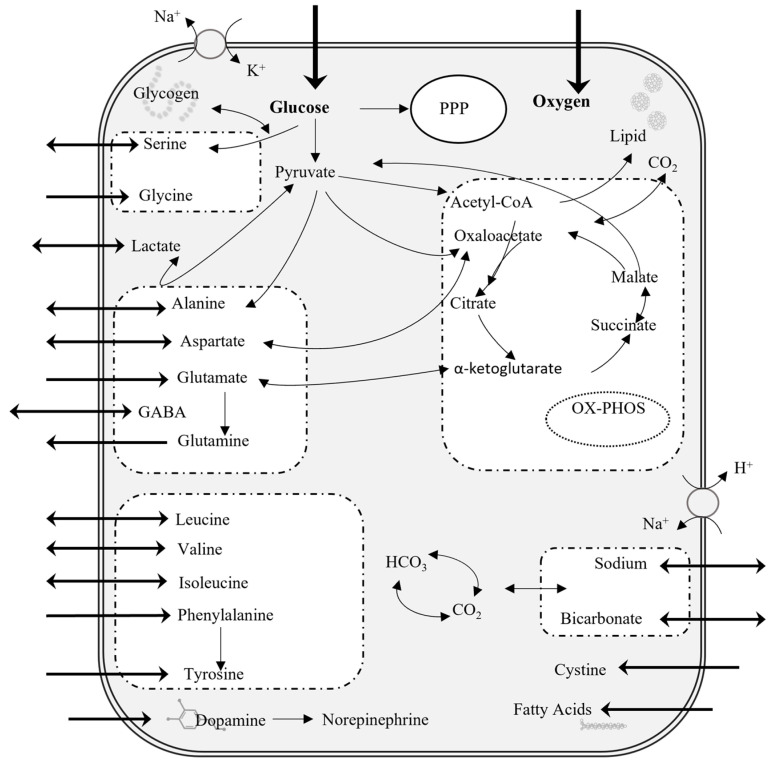
Schematic overview of key metabolic pathways in astrocytes, including glycolysis, the pentose phosphate pathway (PPP), the tricarboxylic acid (TCA) cycle, and oxidative phosphorylation (OxPhos). Major imported substrates are denoted with inward arrows while secreted metabolites are indicated by outward arrows.

One of the key cells in the brain responsible for a variety of functions in the healthy central nervous system (CNS), including regulation of local CNS blood flow, metabolism of lipids and fatty acids, and maintenance of ion and pH homeostasis, is the astrocyte (Figure 1) [21]. Astrocytes take up glucose as an energy substrate for use in glycolysis to produce adenosine triphosphate (ATP) and pyruvate (Figure 1). Astrocytic glycolysis provides the energy necessary for repairing neuronal damage after TBI [22,23]. While glycolysis is the primary metabolic process in astrocytes, they are also capable of mitochondrial oxidative phosphorylation (OxPhos) (Figure 1). In response to TBI, increased astrocyte OxPhos activity reduces oxidative stress on neurons caused by a buildup of reactive oxygen species [23,24]. Astrocyte metabolic phenotypes play a central role in the progression of the wound healing process.

Astrocytes undergo a number of pathophysiological changes that alter their metabolic phenotype under the environmental conditions resulting from TBI. These changes can influence cellular bioenergetics, ultimately contributing to the secondary injury cascades that persist long after the primary injury. Structural changes in astrocytic mitochondria, resulting from fission and fusion, enable these organelles to respond to environmental challenges. Recent work from our group has shown that TBI leads to fragmentation of astrocytic mitochondria, which is known to disrupt proper mitochondrial function [25]. Furthermore, alteration of mitochondrial biogenesis has been observed in association with morphological changes in glial cells, where a shift from OxPhos to glycolysis is accompanied by the activation of glial activation under inflammatory conditions, such as those observed in TBI [26,27]. Thus, understanding how environmental changes in the injured brain can lead to metabolic alterations in astrocytes is a vital step in elucidating mechanisms of glial dysfunction and bioenergetic shifts following TBI.

Computational modeling of astrocyte metabolic activity can serve as a powerful tool for predicting astrocytic responses to environmental changes. To better understand how astrocytes adapt to persistent metabolic stressors of the post-injury environment, we modeled flux distributions under hypoxic and acidic conditions using the previously established Martín-Jiménez et al. astrocyte model [28]. To gain mechanistic insight into these adaptations, we integrated our computational predictions with mass spectrometry-based proteomic data from the frontal cortex of rats three months after closed-head controlled impact. Through this integrative approach, we aim to identify injury-induced shifts in astrocytic metabolism that may underlie chronic dysfunction. By elucidating pathways that are persistently altered, whether as adaptive responses or points of vulnerability, this study seeks to reveal candidate targets for therapeutic intervention and provide a systems-level understanding of astrocyte contributions to long-term outcomes following mild TBI (mTBI).

## 2. Results

### 2.1. Flux Balance Analysis for Astrocytes at Steady State

The previously developed model of astrocyte metabolism [28] was used to simulate normal physiological conditions using two main functions: ATP maintenance and the glutamate–glutamine cycle. The model’s steady state behavior was validated using several experimental observations, including ATP yield per glucose molecule, glycogen storage and metabolism, astrocyte-mediated neurotransmitter recycling, and the higher glycolytic rate of astrocytes compared to their oxidative rate. Astrocytes predominantly rely on glucose, which is broken down into pyruvate, to enter the Tricarboxylic acid (TCA) cycle to drive ATP production [29]. Additionally, some glucose is converted to lactate to meet neuronal energy demands. To confirm active glycolysis in the steady state scenario, key metabolic steps were verified in the model, such as the formation of fructose 2,6-bisphosphate, conversion of pyruvate to lactate, and maintenance of a measurable NAD^+^/NADH ratio. Glycogen, the brain’s largest energy reserve, is almost exclusively stored in astrocytes and plays a vital role in supporting brain function. Thus, flux through both glycogen synthesis and degradation pathways were further evaluated. The model showed active glycogen synthesis, verified by the activity of glycogen synthase (GS), and active glycogen degradation, demonstrated by glycogen phosphorylase (GP) activity. The presence of steady, non-zero flux through both reactions in the model at steady-state confirmed that glycogen storage and mobilization were functional within the model, consistent with the established role of astrocytic glycogen in maintaining brain energy homeostasis.

### 2.2. Change of Flux During Hypoxic, Acidic, or Combined Conditions

#### 2.2.1. Hypoxia

**Figure 2:**
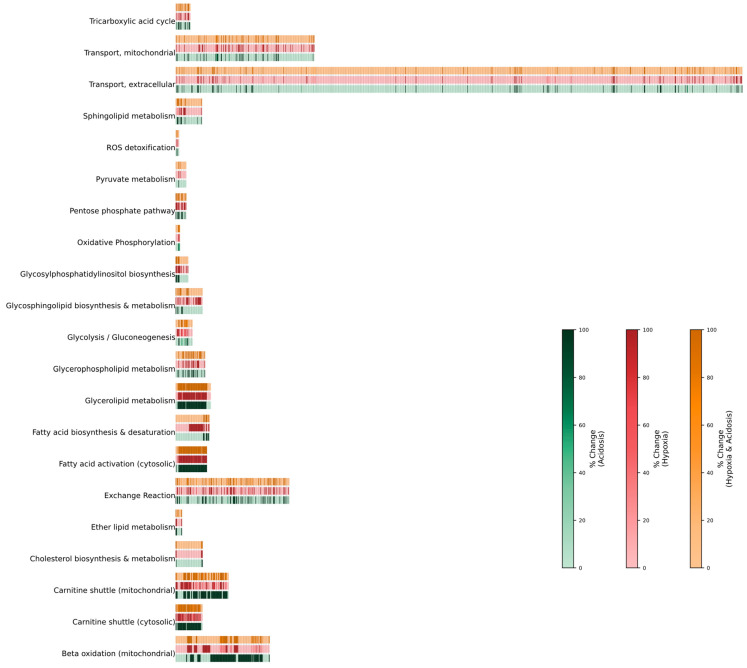
Percent change in reaction fluxes by subsystem and condition. Reactions are denoted as individual blocks with the shading corresponding to the magnitude of percent change. Hypoxic conditions can be found in orange, acidosis is represented as green, and hypoxia + acidosis can be found in red.

To simulate hypoxic conditions, oxygen uptake was limited to a maximum of 2%, and metabolic fluxes were predicted for each reaction. Glycolytic flux increased by 30%, along with an elevation in cytosolic ATP production, with 15 reactions exhibiting robust deviations from steady state levels. Notably, flux through pyruvate dehydrogenase (PDH), which converts pyruvate to acetyl-CoA, was markedly reduced. The model further suggested that astrocytes may utilize both extracellular glucose and intracellular glycogen stores to support the observed increase in glycolysis. Although glucose entered the glycolytic pathway, most of it was derived from glycogen degradation rather than extracellular uptake, accompanied by an increased flux through Pyruvate Kinase M (PKM). OxPhos flux decreased by 48%, with the most pronounced reductions observed in complexes II, IV, and V of the electron transport chain (ETC), leading to a notable reduction in proton generation. This was primarily driven by disruptions in four key reactions. A slight increase in redox activity was also observed, particularly via glutamate dehydrogenase (GDH), which converts α-ketoglutarate to glutamate and NAD^+^. The production of cytosolic NADP^+^ was also enhanced. The fluxes of TCA cycle reactions showed an elevated preference towards the production of mitochondrial carbon dioxide (CO_2_) and glycolaldehyde under hypoxia compared to other conditions. Significant changes were also observed in the fluxes of reactions in the Pentose Phosphate Pathway (PPP) with average fluxes showing its activation. The most pronounced change was observed in phosphoglucomutase 2 (PGM2), which converts ribose-1-phosphate (R1P) to ribose-5-phosphate (R5P). Remarkably, flux through the 4-aminobutyrate exchange increased, suggesting enhanced GABA secretion, while lactate production slightly but unexpectedly decreased. Lastly, although glycerophospholipid and glycerolipid metabolic reactions were robustly affected across all conditions, hypoxia caused substantial increases in fluxes comparable to those seen in the combined condition, mainly through glycerol-3-phosphate 1-O-acyltransferases (GPATs) (Figure 2).

#### 2.2.2. Acidosis

Under acidic conditions, glycolytic flux increased by 28%, along with significant changes in 14 related reactions. Similar to what was observed in hypoxia, this increase was mainly driven by the breakdown of intracellular glycogen rather than uptake of extracellular glucose. Cytosolic ATP production remained similar to that under hypoxia, but mitochondrial ATP production dropped sharply, with OxPhos decreasing by 70%. Surprisingly, complexes I and IV of the ETC exhibited the most pronounced reductions in flux, while reactions associated with complexes III and V showed smaller declines. This led to reduced mitochondrial proton production and an increase in lactate production. A distinctive feature of the acidic condition was the increased flux through the aspartate-glutamate antiporter, which facilitates the exchange of aspartate (exported) and glutamate (imported) as part of the malate–aspartate shuttle. This was accompanied by reduced flux through the TCA cycle, increased activity in the pentose phosphate pathway (PPP), and elevated release of ferrous iron. There was also a noticeable increase in flux through the serine–pyruvate transamination pathway, which produces alanine and hydroxypyruvate. This may reflect an adaptive mechanism for pyruvate disposal and nitrogen buffering. Reactions involved in glycerophospholipid and glycerolipid metabolism showed a remarkable decrease in average flux, while those involved in beta-oxidation of unsaturated fatty acids increased. This increase was mainly driven by the enzymes enoyl-CoA hydratase (ECH), acetyl-CoA C-acyltransferase (AT), acyl-CoA dehydrogenase (ACAD), and 3-hydroxyacyl-CoA dehydrogenase (HADH) (Figure 2). Additionally, there was an increase in potassium (K^+^) exchange and a decrease in sodium (Na^+^) exchange, similar to what was observed under combined stress conditions.

#### 2.2.3. Hypoxia + Acidosis

Notably, under combined hypoxic and acidic conditions, glycolytic flux increased significantly by 98%, while OxPhos activity declined by 89%, with all ETC complexes showing reduced flux. As expected, reactions in the TCA cycle showed remarkable flux decreases whereas those in the PPP were elevated, comparable to all other conditions (Figure 2). Lactate secretion also increased, along with greater flux through the lactate–acetoacetate exchange reaction (L-lactate_c + acetoacetate_e ⇌ L-lactate_e + acetoacetate_c), suggesting a coordinated mechanism to export excess lactate while importing acetoacetate. Compared to hypoxia alone, glycolipid and glycerophospholipid metabolism showed a more pronounced increase, involving enzymes such as glycerol-3-phosphate dehydrogenase (GPDH), GPATs, lysophospholipase, and α-GPC cholinephosphohydrolase (α-GPC). Additionally, glycosphingolipid biosynthesis was elevated (Figure 2). Opposite to what was observed in each condition alone, the combined stress resulted in heighted cytosolic fatty acid activation through acyl-CoA synthetase (ACS) (Figure 2).

### 2.3. Proteomic Insights into Metabolic Pathophysiology Following mTBI

**Figure 3:**
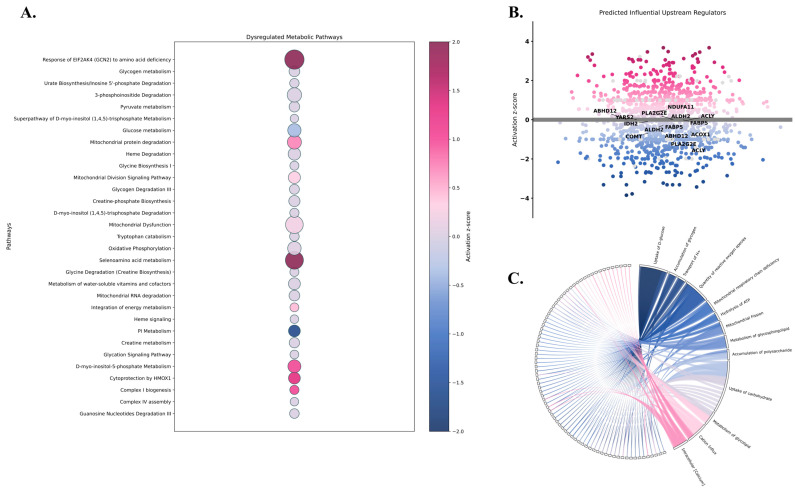
IPA of significantly dysregulated proteins identified in rats 3 months following mTBI. (**A**) Bubble plot illustrating significantly altered metabolic pathways. (**B**) Volcano plot displaying upstream regulators predicted to influence the observed proteomic profile. (**C**) Circos plot depicting the predicted functional metabolic changes. Pathways and regulators having a z-score ≥ 2 were predicted to be activated whereas functional changes were estimated to increase. Those exhibiting a z-score ≤ −2 were projected to be inhibited with the functional changes representing a decrease. Significance was considered with a *p*-value < 0.05.

To better characterize astrocytic metabolic shifts following mTBI, we modeled flux distributions under hypoxic and acidic conditions, two hallmark features of the post-injury microenvironment, and compared these predictions with mass spectrometry-based proteomic data from the frontal cortex of rats 3 months following closed-head controlled impact. IPA analysis revealed widespread mitochondrial dysfunction, including predicted inhibition of ATP synthase and dysregulation across all ETC complexes, consistent with reduced ATP synthase subunit expression and impaired chemiosmotic coupling (Figure 3A). Notably, the Fenton reaction appeared highly activated, with increased hydrogen peroxide and ferrous irons contributing to elevated reactive oxygen species (ROS) production. IPA also indicated enhanced glycolytic activity via activation of hexokinase (HK), Bisphosphoglycerate Mutase (BPGM), and PKM, with the most prominent metabolic bottleneck being the predicted inhibition of glucose-1,6-biphosphate production due to an impairment in PGM2. Pyruvate dehydrogenase kinase 1 (PDK1) overexpression was also noted, with a suggested reduction in pyruvate to acetyl-CoA conversion as well as a decline in isocitrate dehydrogenase (IDH). Notably, phosphorylase was markedly overexpressed (log2FC = 5.581), indicating increased glycogen degradation. Surprisingly, heme oxygenase-1 (HMOX1) cytoprotection was also significantly dysregulated, with IPA anticipating its activation and an increase in cytosolic NADP+ (z-score = 1.342, *p*-value < 0.05). Mitochondrial protein degradation and division were activated, suggesting increased activity of mitochondrial proteases and enhanced mitochondrial fission mediated by dynamin-related protein 1 (DRP1) (Figure 3A). When assessing dysregulations in ion transport, a substantial increase in FXYD domain-containing ion transport regulator 6 (FXYD6) was observed, suggesting heightened Na^+^/K^+^-ATPase pump activity. Similar trends were also noted in the projected elevation of V-ATPase activity due to V-type proton ATPase subunit E1 (ATP6V1E1) and Aspartate Beta-Hydroxylase (ASPH) overexpression.

IPA upstream regulator and causal network analysis further predicted the dysregulation of aldehyde dehydrogenase 2 (ALDH2), acyl-CoA oxidase 1 (ACOX1), ATP citrate lyase (ACLY), Abhydrolase Domain Containing 12 (ABHD12), Fatty Acid-Binding Protein 5 (FABP5), Phospholipase A2 Group IIE (PLA2G2E), NADH:Ubiquinone Oxidoreductase Subunit A11 (NDUFA11), tyrosyl-tRNA synthetase (YARS2), catechol-O-methyltransferase (COMT), and isocitrate dehydrogenase 2 (IDH2) (Figure 3B).

Lastly, diseases and functions related to metabolic dysregulation were revealed as depicted in Figure 3C. Of relevance, functional predictions suggested increased glucose uptake, decreased glycogen stores, enhanced glycosphingolipid metabolism, and impaired carbohydrate storage. Furthermore, mitochondrial disorder and mitochondrial respiratory chain deficiency showed a statistical likelihood of being associated with the molecular landscape. Interestingly, a reduction in astrocytosis and cortical astrocyte survival was predicted, suggesting long-term astrocytic vulnerability post-injury. Ionic perturbations were also noted showing disruptions in influx and transport of H^+^, Ca^2+^, and additional cations.

### 2.4. Bridging Predicted Metabolic Fluxes with Proteomic Alterations

**Figure 4:**
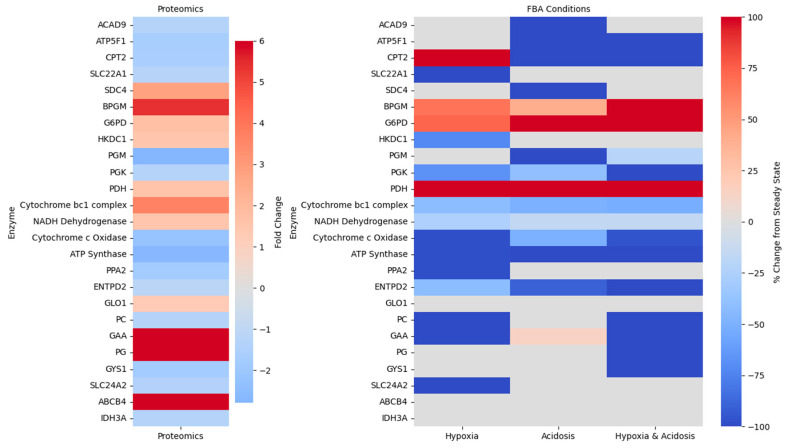
Split heatmap of proteomics and FBA results. The left panel shows fold changes for each essential enzyme. The right panel displays percent changes in predicted reaction fluxes, relative to steady state, under Hypoxia, Acidosis, and combined Hypoxia and Acidosis conditions. Each row corresponds to a single enzyme–reaction pair, grouped and ordered by metabolic subsystem to emphasize pathway-level patterns. Color intensity indicates the direction and magnitude of change: red for upregulation, blue for downregulation, and white representing no change.

Metabolic fluxes simulated under hypoxic, acidic, and combined stress conditions were compared with key DEPs identified post-mTBI (Figure 4). All conditions exhibited consistent perturbations in the same glycolytic reactions, indicating a strong and reliable impact on glycolysis. This was supported by proteomic evidence of glucose-6-phosphate dehydrogenase (G6PD) overexpression and PGM2 reduction, suggesting elevated PPP activity, increased glycolysis, and reliance on glycogen stores. Across all conditions, metabolic fluxes were also consistent with the overexpression of PDK1, suggesting a coordinated shift away from oxidative metabolism toward glycolysis via inhibition of pyruvate entry into the TCA cycle. Consequently, IDH reactions showed no detectable flux, specifically under hypoxic and combined conditions, aligning with reduced IDH expression. Strikingly, the OxPhos proteomic profile aligned with flux analysis across all conditions, revealing significant dysregulation spanning all ETC complexes. This was reflected in reduced cytochrome c oxidase activity and decreased flux through reactions associated with ATP synthase subunits, based on gene-reaction associations. Likewise, carnitine palmitoyltransferase 2 (CPT2), which plays an essential role in energy production and lipid biosynthesis, was significantly decreased post-injury, with flux predictions suggesting acetyl-CoA buffering via the carnitine system.

IPA further predicted inhibition of succinate-to-fumarate conversion, consistent with succinate accumulation under hypoxia. Hypoxicspecific shifts also included an increase in mitochondrial carnitine export, corresponding with the observed molecular downregulation in the solute carrier family 22 member 1 (SLC22A1). Proteomic dysregulations aligned with acidosis-induced changes, including a reduction in 3-hydroxyanthranilate 3,4-dioxygenase (HAAO) expression, which indicated a shift away from 2-amino-3-carboxymuconic semialdehyde synthesis. Hexosaminidase subunit alpha (HEXA) was significantly overexpressed following injury, with increased flux through HEXA-catalyzed reactions in hypoxic and combined conditions, suggesting oxygen-dependent glycolipid metabolism. Interestingly, these reactions shifted away from glycolipid and glycosphingolipid metabolism, during acidic conditions.

Upstream regulator and causal network analysis predicted downregulation of ACOX1, with its corresponding reactions showing flux increases in hypoxic and acidic conditions and a decrease in the combined condition. Hypoxia-specific flux increases in CO_2_ and glycolaldehyde production, along with elevated glycolaldehyde oxidation, pointed to a compensatory response to aldehyde accumulation, supported by ALDH2 dysregulation. Post mTBI, dysfunction of FABP5 was also inferred, with substantial flux alterations observed in decanoic acid transport across all stress conditions. FBA also revealed a coordinated suppression of lipid mobilization under hypoxic and combined stress conditions, evidenced by decreased flux through phosphatidylcholine and glycerophosphocholine hydrolysis reactions associated with the lipid droplet (LD) pool. These reactions are critical for generating free fatty acids and lysophospholipids, suggesting a downregulation of phospholipid turnover and fatty acid release. This aligns with IPA-based predictions indicating reduced expression of ABHD12 and PLA2G2E, key lipases involved in fatty acid metabolism.

## 3. Discussion

The combinatorial analysis of proteomic data from mTBI and predicted metabolic fluxes in astrocytes under hypoxic and acidic conditions provides unique insights into the regulatory and functional adaptations of brain metabolism following injury. This study identifies overlapping dysregulations as consistent metabolic vulnerabilities, while the divergences may be leveraged for targeted intervention. Functionally, FBA revealed increased activity in glycolysis, the PPP, and glycerolipid and glycerophospholipid metabolism across all conditions. Conversely, OxPhos and the TCA cycle consistently showed reduced flux. The transport and exchange reaction subsystems, along with those involved in the carnitine shuttle, exhibited the most substantial flux alterations. At the regulatory level, proteins chronically dysregulated after mTBI were most significantly associated with mitochondrial dysfunction, particularly impairments in ETC biogenesis and OxPhos. While early glycolytic proteins were activated, the data also suggested inhibition of glucose and pyruvate metabolism, alongside increased glycerolipid/glycerophospholipid activity. This profile implies glycolytic rigidity and an increased reliance on the PPP, supported by increased G6PD expression. The upregulation of PDK1, alongside corresponding flux changes, suggests chronic inhibition of pyruvate dehydrogenase, limiting TCA cycle input. This integrative analysis also revealed core astrocytic vulnerabilities in the ETC, particularly cytochrome c oxidase dysfunction and impairment in the proton motive force. Proteomic data identified the EIF2AK4 response to amino acid deficiency as one of the most significantly activated pathways. This nutrient-sensing mechanism conserves amino acids and facilitates ammonium detoxification, aligning with FBA findings of elevated flux through ammonium-assimilating and glutamine-producing reactions. Specifically, robust activity in the conversion of α-ketoglutarate and ammonia to glutamate, followed by ATP-dependent glutamine synthesis, was observed across all conditions. These fluxes, though partly model-driven, are reinforced by proteomic evidence of upstream pathway activation, underscoring their relevance as survival mechanisms.

Although the carnitine system was not significantly dysregulated at the proteomic level, aside from reduced CPT2 expression, its role in post-injury metabolism is well documented in the literature [30]. In our study, protein expression alone did not indicate activation or inhibition of the carnitine shuttle. However, metabolic flux analysis revealed substantial rerouting of lipid substrates through this pathway across all simulated conditions, even under environmental stress. This functional engagement, despite mitochondrial and proteomic constraints, suggests an alternative role for the carnitine shuttle, underscoring its importance in preserving metabolic flexibility after injury and highlighting it as a promising target for in vivo intervention. One of the most compelling therapeutic opportunities identified through integrative flux analysis involves ATP salvage via R5P. Although IPA revealed activation of purine catabolism and P2Y purinergic receptor signaling, the specific role of purine salvage in energy buffering was not emphasized. Despite reduced OxPhos and TCA activity, astrocytes continued to meet their energetic demands, in part through noncanonical ATP-producing pathways, particularly phosphotransfer reactions involving R5P. This mechanism remained active under all modeled stress conditions, suggesting purine metabolism contributes to survival under chronic mitochondrial dysfunction. This finding is in agreement with previous studies suggesting that targeting this pathway may support cellular energetics [31].

Previous preclinical studies have reported dysregulation of key proteins involved in glycolysis, OxPhos, ATP synthesis/transport, and the TCA cycle following TBI. Specifically, IPA-predicted ATP synthase suppression aligns with prior findings of downregulated ATP synthase subunits in the CA3 subregion of the hippocampus four days post-TBI [32]. Conversely, IPA predicted enhanced glycolysis in the injured frontal cortex, including increased hexokinase activity and inhibited PDH complex activity. However, Wu et al. reported downregulation of both hexokinase-1 and the beta subunit of the PDH complex [32]. NDUFA11 was predicted to be significantly dysregulated upstream of the proteomic changes in our TBI model. Similarly, NDUFA9 and NDUFA10 were downregulated in the hippocampus in the same study, indicating OxPhos dysfunction [32]. Wu et al. also reported inhibition of canonical pathways for glycolysis, OxPhos, and pyruvate metabolism [32]. Similarly, TBI-induced inhibition of OxPhos and activation of glycogen degradation have been reported acutely in the prefrontal cortex [33], consistent with our findings of decreased OxPhos flux across simulated conditions and increased reliance on glycogen breakdown for energy metabolism. Chronic proteomic alterations in energy metabolism were also observed by Ojo et al., including disruptions in mitochondrial bioenergetics, which support our observations [34].

While this integrative analysis provides valuable insights into potential metabolic adaptations following mTBI, there are important limitations. FBA infers fluxes based on network topology, defined constraints, and objective functions. In this study, ATP maintenance and glutamate/glutamine cycling were prioritized. Consequently, the predicted flux distributions reflect theoretically optimal or compensatory routes rather than actual in vivo activity of injured astrocytes which may be operating suboptimally. Additionally, the proteomic analysis did not account for post-translational modifications, enzyme activity states, or metabolite concentrations. Notably, the proteomic data were derived from frontal cortex tissue containing all cell types, whereas the FBA model focused solely on astrocytic metabolic responses. Thus, the dysregulated proteins and pathways may reflect contributions from multiple cell types, not just astrocytes, making it difficult to pinpoint astrocyte-specific proteomic changes. Lastly, this work focused on examining the simulated effects of hypoxia and acidosis on metabolic activity, as these conditions are both relevant to the post-TBI microenvironment. However, other contributors to the post-TBI microenvironment, including neuroinflammation and oxidative stress, can also influence metabolic activity. The importance of inflammation and oxidative stress are a direction of future work as this model allows for tailoring of different astrocytic pathological states.

The consistent overlap between flux changes and proteomic data, especially in glycolysis, OxPhos, and glutamate/glutamine cycling, suggests conserved adaptive mechanisms employed by astrocytes under injury and induced stress. These common signatures may reflect core survival strategies, offering insight into metabolic priorities and potential biomarkers for injury adaptation or recovery. Conversely, the discrepancies observed between flux predictions and proteomic findings, particularly in ATP-maintenance pathways, highlight metabolic vulnerabilities. These gaps indicate that while the metabolic network has the theoretical capacity to maintain homeostasis, key enzymes or regulatory mechanisms may be insufficiently activated under injury conditions. Such weaknesses offer avenues for targeted intervention through enzyme activation, pathway modulation, or gene therapy. Ultimately, comparing modeled metabolic outputs with in vivo proteomic data derived from the brains of TBI-exposed rodents provided important insights into the alignment and divergence between predictions generated by the FBA model and molecular observations in vivo.

## 4. Materials and Methods

### 4.1. Flux Balance Analysis

Flux balance analysis was used to provide insights into the biological consequences of astrocyte metabolism in hypoxic and acidic conditions. Modifying a previously reconstructed flux balance model of astrocyte metabolism [28], reactions were assessed for biological relevance and constraints were applied to respective reactions to simulate normal physiological conditions (Table 1). The metabolic requirement of astrocytes supports a glycolytic profile, displaying elevated glycolytic rates and lower oxidative rates. Therefore, the multi-objective functions for the metabolic requirements of astrocytes were defined as:

1. ATP Maintenance prioritizes ATP production to meet the energetic demands of the cell.

ATP_c_ + H_2_O_c_ → ADP_c_ + Pi_c_

2. Glutamate input and glutamate output due to the essential role of astrocytes in neurotransmitter recycling.

Glutamate_e_ + Glutamine_c_ → Glutamate_c_ + Glutamate_e_

The multi-objective function was then optimized for our study, focusing on selective pathways essential for brain metabolic homeostasis in order to address the distinct metabolic differences between astrocytic profiles in each condition. Perturbations were then applied by varying the lower and upper bounds of the corresponding reactions. To simulate hypoxic conditions, bounds to the oxygen exchange reaction ‘Ex_O_2__e’ were constrained by reducing oxygen uptake rates, allowing 2% of oxygen into the system. For acidosis, astrocytes have a strong inward electrochemical gradient for H+ due to their highly negative membrane potential. This gradient energetically favors the influx of H+ into the cytosol. As a result, astrocytes must continually export acid equivalents from the cytosol to the extracellular space to counteract intracellular acidification and maintain their pH within the physiological range. Thus, we adjusted the bounds of the Na^+^/H^+^ exchanger and CO_2_/bicarbonate system to result in a reduction of astrocyte buffering capacity. Flux balance analysis was performed on each condition using CNApy, and the resulting flux values along with their associated metabolites were exported for further analysis. To identify which reactions were strongly affected by each condition, we analyzed changes in reaction fluxes relative to the steady state. A threshold of 10% was applied, where any reaction with a relative change exceeding this threshold was flagged as exhibiting a robust change. For these flagged reactions, we identified the enzymes responsible for catalyzing these reactions, providing us insight into those most impacted under the given condition.

### 4.2. Experimental Proteomic Data Acquisition

For experimental validation, predictions generated by the FBA model were compared to mass spectrometry-based proteomics generated from a previous study [50]. All procedures were conducted following approval from the Virginia Tech Institutional Animal Care and Use Committee. Animals were acclimated for two weeks on a 12-12-hour dark-light cycle. Rats were maintained at 70° F and 70% humidity and had ad libitum access to food and water. Enrichment included wooden blocks and sticks placed in each cage. In brief, adult (12 weeks old) wildtype male Sprague Dawley rats (Envigo; Dublin, VA, USA) (*n* = 6 total; 3 per group) underwent closed-head controlled impact or sham procedures to induce a mild TBI. Animals were randomly assigned to the sham or TBI groups using a random number generator, and potential confounders were minimized by ensuring all cages were evenly distributed across shelves to reduce location-related variability. A priori exclusion criteria consisted of skull fracture, mortality, or loss of >20% of pre-injury body weight as these constituted the humane endpoints of the study. Animals were monitored weekly following injury for signs of pain or distress. No animals were excluded from the study. All procedures were conducted in a vivarium procedure room. Closed-head controlled impact was conducted using an electromagnetic impactor (Impact One, Leica Biosystems, Buffalo Grove, IL, USA; RRID:SCR_025114) with a tip diameter of 5 mm. The impact was delivered at a velocity of 6 m/s, a depth of 5 mm, and a dwell time of 300 ms over the right parietal cortex. Animals were anesthetized under 4% isoflurane during impact procedures. Sham animals were anesthetized but did not undergo impact. Animals were euthanized at three months post-injury, and the right (ipsilateral) frontal cortex was harvested, rinsed in ice-cold saline, and snap-frozen on dry ice. Protein lysates (*n* = 3 samples per group) were prepared, digested with trypsin, and analyzed using LC-MS/MS (Orbitrap Fusion Lumos, Thermo Fisher Scientific, Waltham, MA, USA) as described previously [51,52]. LC-MS/MS analysis was conducted by a researcher blinded to the experimental grouping of the animals. Protein abundances were normalized to total peptide intensity, and differentially expressed proteins (DEPs) were identified based on fold-change thresholds (|FC| ≥ 1.2), adjusted *p*-value (*p* < 0.10), and false discovery rate (FDR ≤ 0.05).

### 4.3. Ingenuity Pathway Analysis of Differentially Expressed Proteins

To identify metabolic shifts associated with environmental stressors post-injury, differentially expressed proteins (DEPs) were subjected to functional enrichment and pathway analysis using Ingenuity Pathway Analysis (IPA), Gene Ontology (GO), Kyoto Encyclopedia of Genes and Genomes (KEGG), and Reactome tools. These analyses aimed to characterize the metabolic relevance of the DEPs and predict the potential biological consequences. IPA was used to identify canonical pathways, upstream regulators, and causal networks, with those showing a *p*-value ≤ 0.05 considered significantly dysregulated. Additionally, disease and functional analyses were conducted to assess the molecular changes underlying metabolic dysregulation. A z-score ≥ 2 indicated a predicted increase in the associated pathway or function, while a z-score ≤ −2 indicated a predicted decrease. Only those disease and functional associations with a *p*-value ≤ 0.05 were included, highlighting the statistical likelihood that a particular disease or function was significantly linked to the observed proteomic profile.

## Figures and Tables

**Table 1:** Astrocyte uptake and release rates for different metabolites.

Metabolite	Metabolic Rate µmol/g/min	References
Acetoacetate	0.012	[35]
Glutamine	0.500	
Asparagine	0.0037	[36]
Ornithine	0.0031	[37]
Valine	0.0018	[38]
Serine	0.0016	[37]
Linoleate	0.0011	[39]
Threonine	0.0008	[40]
Isoleucine	0.0004	[41]
Methionine	0.0017	[42]
Tyrosine	0.0017	[36]
Arginine	0.0025	[43]
Histidine	0.0025	[44]
Cystine	0.0045	[45]
Lysine	0.011	[46]
Leucine	0.0145	[46]
Glutamate	0.232	[47]
Acetate	0.0013	
Glycine	0.0053	[47]
Proline	0.0066	[48]
Alanine	0.0079	
Glucose	0.19	[40,49]
CMR_O_2_	0.530	[48]
CMR_CO_2_	0.515	[48]

## Data Availability

The raw data supporting the conclusions of this article will be made available by the authors on request.

## Abbreviations

The following abbreviations are used in this manuscript:

TBI	Traumatic brain injury
mTBI	Mild traumatic brain injury
CNS	Central nervous system
TCA	Tricarboxylic acid
PPP	Pentose Phosphate Pathway
OxPhos	Oxidative Phosphorylation
ATP	Adenosine triphosphate
ETC	Electron transport chain
CO_2_	Carbon dioxide
FBA	Flux balance analysis
DEPs	Differentially expressed proteins
FC	Fold change
GO	Gene ontology
KEGG	Kyoto Encyclopedia of Genes and Genomes
IPA	Ingenuity Pathway Analysis
